# Assessment of Long-Term Hematologic Effects in Differentiated Thyroid Cancer Patients Treated with Radioactive Iodine

**DOI:** 10.4274/tjh.galenos.2021.2021.0092

**Published:** 2021-12-07

**Authors:** Bircan Sönmez, Özlen Bektaş, Nergiz Erkut, Mehmet Sönmez

**Affiliations:** 1 Karadeniz Technical University Faculty of Medicine, Department of Nuclear Medicine, Trabzon, Turkey; 2 Karadeniz Technical University Faculty of Medicine, Department of Hematology, Trabzon, Turkey

**Keywords:** Radioactive iodine, Thyroid cancer, Cytopenia, Hematologic malignancy, Long-term hematologic effects, Thrombocytopenia, Neutropenia

## Abstract

**Objective:**

Radioactive iodine (RAI) therapy may cause hematologic abnormalities. The aim of this study is to evaluate long-term hematologic effects in differentiated thyroid cancer (DTC) patients after RAI therapy.

**Materials and Methods:**

A total of 1389 patients with DTC who were treated with RAI were retrospectively evaluated. Complete blood cell counts before RAI therapy and at last follow-up and hematologic malignancy development were obtained from the electronic records.

**Results:**

In the long-term analysis, thrombocytopenia and lymphopenia were observed significantly in patients over 60 years of age. Thrombocytopenia was observed more frequently in men. Leukopenia, thrombocytopenia, and lymphopenia were observed significantly with doses of >175 mCi. Thrombocytopenia and lymphopenia were observed significantly with multiple dose administration. Higher frequencies of anemia, thrombocytopenia, leukopenia, neutropenia, and lymphopenia were found in patients with advanced-stage disease. However, patients with advanced-stage disease had higher doses and more multiple doses than patients with early-stage disease. The rate of hematologic malignancy was found to be higher than in the general population.

**Conclusion:**

We suggest that cytopenia be surveyed more carefully in patients older than 60 years of age. The most important risk factor for lower platelets after RAI therapy is male gender. Clinically, the most important predictor for cytopenia is advanced disease stage, which is related to the combined effects of applied high dose activity, multiple dose applications, and high tumor burden.

## Abstract (tr)

**Amaç:**

Radyoaktif iyot (RAİ) tedavisi hematolojik anormalliklere yol açabilir. Bu çalışmada, diferansiye tiroid kanserli (DTC) hastalarda RAİ tedavisi sonrası uzun dönemli hematolojik etkilerin değerlendirilmesi amaçlanmıştır.

**Gereç ve Yöntemler:**

Bin üç yüz seksen dokuz RAİ ile tedavi edilmiş DTC’li hasta retrospektif olarak değerlendirildi. Tedavi öncesi, son takip ve hematolojik malignite geliştikten sonra ölçülen tam kan sayımları elektronik kayıtlardan elde edildi.

**Bulgular:**

Uzun dönemli analizde trombositopeni ve lenfopeni özellikle 60 yaş üstü bireylerde gözlendi. Trombositopeni erkek hastalarda daha sıktı. Lökopeni, trombositopeni ve lenfopeni >175 mCi dozlarda anlamlı oranda artmıştı. Trombositopeni ve lenfopeni çoklu doz uygulaması olanlarda anlamlı oranda izlendi. Anemi, trombositopeni, lökopeni, nötropeni ve lenfopeni, ileri evre hastalıklı hastalarda daha sıktı. Ancak ileri evre hastalığa sahip hastalar, erken evrelilere göre hem daha yüksek doz hem de çoklu doz tedavi almışlardı. Hematolojik malignite oranı genel popülasyondan daha yüksek oranda idi.

**Sonuç:**

Özellikle 60 yaş üstü hastalar sitopeniler açısından daha yakın izlenmelidir. RAİ tedavi sonrası en önemli risk faktörü erkek cinsiyettir. Klinik olarak sitopeniler için en önemli belirleyici ileri evre hastalıktır, ki bu da, daha yüksek doz ve çoklu doz tedavi uygulanmış olmasının ve daha fazla tümör yükünün kombine etkisi ile ilişkilidir.

## Introduction

Radioactive iodine (RAI) therapy is a commonly used therapeutic option for patients with differentiated thyroid cancer (DTC) for ablation of residual thyroid tissue after thyroidectomy or for the treatment of recurrent and metastatic disease [1]. RAI doses of 30-100 mCi are used for ablation of residual thyroid tissue. Higher and repeated doses (up to 200 mCi for single doses and 600 mCi for cumulative doses) can be applied for locoregional recurrence or metastatic disease [1,2,3].

After RAI therapy, side effects such as sialadenitis, nasolacrimal duct obstruction, keratoconjunctivitis, amenorrhea, and hematologic abnormalities can be observed in the first few months [4]. Most of these early side effects are usually temporary and have little long-term clinical significance. However, several meta-analyses have reported an increased incidence of second primary malignancies (SPMs) in patients with DTC treated with RAI [5,6,7].

Temporary anemia, leukopenia, and thrombocytopenia may occur within the first month after a single RAI therapy [8]. Several studies reported improvement in complete blood cell (CBC) counts at 6 months to 1 year after treatment [9,10], while in other studies, the decrease in leukocytes [11], platelets [8,11,12], and lymphocytes [12] persevered.

In our study, we aimed to evaluate the potential long-term effect of RAI on the hematologic system in patients with DTC who received RAI therapy.

## Materials and Methods

A total of 1389 patients who were treated with RAI therapy after total thyroidectomy during 2005-2018 were retrospectively evaluated. Exclusion criteria were i) bone marrow infiltration, ii) receiving external beam radiotherapy and/or chemotherapy at any time, iii) concurrent or pretreatment hematologic malignancies, and iv) patients developing solid cancer. The inclusion criterion was the development of hematologic malignancy after RAI therapy. At the time of the initial treatment, all patients were staged according to the 8^th^ version of the AJCC TNM classification. Each patient was categorized according to receiving single or repeated doses of RAI therapy.

Radioiodine therapy were performed with fixed doses as follows: ^<^100 mCi (dose I) for remnant ablation, 125-150 mCi (dose II) for those with lymph node metastasis, and ^>^175 mCi (dose III) for metastatic disease. Levothyroxine (L-T4) was discontinued 4-6 weeks and triiodothyronine (L-T3) was discontinued 2 weeks before RAI therapy. A low-iodine diet for 2 weeks was recommended to all patients before RAI therapy. L-T4 treatment was restarted 48 h after RAI therapy. Two or more doses were applied to the patients with locoregional recurrence and/or distant metastasis. CBC results before RAI therapy and at the last follow-up and the development of hematologic malignancy were obtained from the electronic records. The criteria for anemia were hemoglobin of <12 g/dL in women and <13.0 g/dL in men, while neutropenia was defined at a level of <1500/µL, leukopenia at <4000/µL, thrombocytopenia at <150x10^9^/µL, and lymphopenia at <1000/µL.

This study was approved by the Local Ethics Committee of Karadeniz Technical University.

### Statistical Analysis

Data analysis was performed using SPSS 22.0 for Windows. Descriptive statistics were shown as mean ± standard deviation or median (minimum-maximum) for continuous variables and as number of cases (%) for categorical variables. Significance between baseline and last follow-up laboratory values was analyzed with the paired samples t-test. Significance between groups was analyzed with the Mann-Whitney U test when the number of independent groups was two and with the Kruskal-Wallis test when the group number was greater than two. If there was a significant difference in the outcome of the Mann-Whitney U or Kruskal-Wallis tests, post hoc tests were used to determine the variables responsible for this significance. Correlation between continuous variables was examined with Spearman’s correlation test for non-parametric variables and with Pearson’s correlation analysis for parametric variables. Odds ratios were analyzed with binary and multinominal logistic regression. Values of p<0.05 were considered statistically significant.

## Results

While 263 patients (18.9%) were male, 1126 (81.1%) were female. Mean age was 47.44±12.52 (range: 8-82) years. Mean follow-up period and time of last follow-up CBC results was 60.47±36.60 (range: 6.67-386.33) months. Baseline characteristics of patients are summarized in Tables 1 and 2.

Upon comparing the differences between baseline and last follow-up hematologic values, a significant decrease was observed in hemoglobin and platelets at the last follow-up (p=0.000, p=0.012). The change between baseline and last follow-up lymphocyte values was significant when patients with hematologic malignancies were excluded (p=0.002) (Table 3).

A negative correlation was found between age and white blood cell (WBC) count, neutrophils, and platelets (r=-0.094, p=0.000; r=-0.093, p=0.001; r=-0.126, p=0.000, respectively) and a positive correlation was found between age and hemoglobin according to the last follow-up laboratory values of the patients (r=0.076, p=0.004).

It was observed that platelets were significantly lower in patients over 60 years of age (p<0.001). All series in women except platelets and lymphocytes were found to be significantly lower than in men. It was observed that lymphocytes decreased significantly when the dose was increased. Dose 1 with dose 2 and dose 1 with dose 3 made the differences (p=0.001 and p=0.008, respectively). Hemoglobin, platelets, and lymphocytes also decreased as the stage increased. Stages I and IV for hemoglobin, platelets, and lymphocytes (p=0.041, p=0.001, p=0.006, respectively) and stages II and IV for hemoglobin (p=0.046) made the differences (Table 4).

Before treatment, anemia was present in 223 patients (16.1%), leukopenia in 33 patients (2.4%), thrombocytopenia in 21 patients (1.5%), neutropenia in 10 patients (0.7%), and lymphopenia in 20 patients (1.4%). When the last follow-up cytopenia status of the patients was examined, it was observed that rates of thrombocytopenia and lymphopenia were significantly higher in patients over 60 years of age (p=0.013, p=0.018, respectively). Males had thrombocytopenia more often than females (p=0.006). Higher doses were a risk factor for leukopenia, thrombocytopenia, and lymphopenia and the risk increased with RAI of ^>^175 mCi. The frequencies of thrombocytopenia and lymphopenia increased with repeated doses (p=0.003, p=0.003, respectively). Cytopenia was more frequent in all 5 series in stage >II (stage IV for this study, since there were no stage III patients in the study group) compared to the earlier stages (Table 5).

Acute myeloid leukemia (AML) developed in 0.2% cases (n=3), chronic lymphocytic leukemia (CLL) in 0.3% (n=4), and myelodysplastic syndrome (MDS) in 0.1% (n=2) (Table 6). The mean time until the development of hematologic malignancy was 38.13±33.82 (range: 5.46-104.45) months. When hematologic malignancies were evaluated according to their prevalence in the world, the prevalence in our sample was found to be higher than that of the general population.

## Discussion

RAI therapy is one of the standard treatments for DTC. Orally administered RAI diffuses into the circulation through the gastrointestinal tract. The whole body is exposed to highly energetic b- and g-radiation during its transport to, accumulation in, and destruction of thyroid tissue and the urinary excretion of the RAI [13]. Cell renewal, apoptosis, and redistributions of the hematopoietic cells are affected by this radiation [14].

In this study, we have evaluated the long-term hematologic complications of RAI therapy in DTC patients. When the differences between pretreatment and last follow-up laboratory values were examined, it was observed that final hemoglobin and platelets were significantly lower than the baseline levels regardless of gender, applied activity, and number of applications, which suggested that RAI therapy can decrease blood cell levels regardless of cytopenia. There was no significant change between the baseline and last follow-up lymphocyte values when patients with hematologic malignancies were included. We considered that patients with CLL could have increased last follow-up mean lymphocyte counts and repeated the statistical analysis without patients with hematologic malignancies. It was observed that lymphocyte counts were significantly lower when hematologic malignancies were excluded (p=0.002). In the literature, one of the most common complications in long-term follow-up after RAI therapy is a decrease of platelets [11,15]. Lymphocytes are the most radiosensitive of all hematologic cells [14]. Granulocytes/monocytes are also more sensitive than erythroid series [13]. However, a decrease in the erythroid series is an expected finding. Molinaro et al. [11] did not observe a change in hemoglobin during 1 year of follow-up, while Schober et al. [15] showed that thrombocytopenia and erythrocytopenia were the most common types of cytopenia in a period of 65 months. Sönmez et al. [8] showed a significant decrease in hemoglobin during 1 year of follow-up.

We observed that hemoglobin levels were higher among patients of older age. As age increases, the number of women entering menopause also increases. Considering that women constituted a significant majority of our cases (81%), this may explain the hemoglobin increase. We found that platelet counts were significantly lower and rates of clinical thrombocytopenia and lymphopenia were significantly higher in patients over 60 years of age. A decrease in platelets with age after RAI has been previously reported [16]. The decrease in bone marrow reserve with age may explain the thrombocytopenia and lymphopenia.

We found gender-specific differences for certain types of cells. Mean hemoglobin, WBC count, and neutrophils were lower in women than in men. The reason for the difference in hemoglobin is that the normal range of hemoglobin of women is lower than that of men. When the presence of anemia was evaluated, no difference was observed between men and women. However, thrombocytopenia was observed more frequently in men than in women, which indicates that platelets in men are more sensitive to RAI therapy than in women. Prinsen et al. [16] reported more frequent thrombocytopenia in men after RAI. Hu et al. [12] showed in a study with 385 patients that there was a decrease in WBC and lymphocyte counts without gender difference and a greater decrease in platelets in women than in men during the 6-month follow-up. Fewer cases and the shorter follow-up period of that study may be the main reasons for the difference from our results.

We observed that cytopenia occurred more frequently with higher applied activity of RAI. Leukopenia, thrombocytopenia, and lymphopenia occurred more frequently at RAI doses of ^>^175 mCi. A similar result was previously reported about thrombocytopenia and excessive cumulative dose [16]. Padovani et al. [17] showed low hemoglobin and platelets, especially at > 250 mCi. When we evaluated the effects of multiple doses, we observed that thrombocytopenia and lymphopenia were more common.

In our study, platelet and lymphocyte counts were found to be lower in patients with stage IV disease. Since there were no patients with stage III in our study, an evaluation could not be performed with that stage. Additionally, as the stage increased, an increase in the frequency of cytopenia was detected in all series. However, it must be noted that advanced-stage patients received higher dose activities and more multiple-dose therapy. It is known that after RAI treatment, the blood RAI concentration shows a diphasic course. In the first 24-48 h, a rapid decrease in inorganic ^131^I in the blood is observed due to rapid clearance by the kidneys, functional tumor tissue, and remaining thyroid tissue. In the next 2-10 days, a protein-bound ^131^I peak is observed due to release by residual thyroid tissue and/or functional tumor tissue. This can cause RAI to be carried in the body for days [18]. Therefore, higher tumor burden may cause large amounts of RAI to be released into the circulation with a long duration of stay and, as a result, increased bone marrow toxicity may occur. It has been reported that thrombocytopenia is observed more frequently in individuals with large tumor masses [16]. The strongest results in our study were obtained for disease stage and cytopenia. We suggest that the clinical effect of advanced disease stage on cytopenia was due to the combined effect of three factors: higher applied activity, multiple RAI administration, and higher tumor burden.

The incidence of AML is 1.6-2.8 cases per 100,000 in men and 1.0-2.2 cases per 100,000 in women [19], and the incidence of CLL is approximately 4.2 cases per 100,000 people in the world population [20]. Acute and chronic leukemias have been reported after RAI therapy [18,21]. The incidence of leukemia increases with >600 mCi activity, >45 Gy, and treatment with short intervals [11,22]. Leukemia has been reported very rarely at <300 mCi activity [11]. Cumulative dose was reported to be the strongest risk factor for leukemia [22,23], aplastic anemia, and MDS [23]. When hematologic malignancies were evaluated according to their incidence in the world, the incidence in our sample was found to be higher than that of the general population. Regarding hematologic malignancies, no relationship was observed with age; furthermore, diagnoses of MDS, AML, and CLL were more frequent in the low-dose RAI group including single dose administration and early-stage disease. We did not observe the reported risk factors for hematologic malignancies in our patient group. Although RAI is known to be a risk factor for leukemia, SPMs cannot be ruled out. Karaköse et al. [24] detected 70 SPMs in 1196 patients with thyroid cancer. Thirty-two of them were diagnosed with a second malignancy after thyroid cancer and 38 of them were diagnosed with another malignancy before thyroid cancer. RAI treatment was given to 25 patients in each group. SPM independent of RAI was detected in 45 patients (3.8%) [24]. The incidence of the malignancy in Turkey is 0.2%. As to be expected, it was reported that the prevalence of SPM was increased by 20 times in patients with thyroid cancer [24]. Silva-Vieira et al. [25] detected SPMs in 4.8% of patients with thyroid cancer who did not receive RAI treatment. The risk of leukemia is higher in patients with thyroid cancer, and especially in those treated with RAI. The 5- to 10-year absolute leukemia development risk is 0.23%-0.26% in patients with thyroid cancer treated with RAI [26]. The results of our study are similar as the AML rate was 0.22% and CLL was 0.28% in patients receiving RAI.

### Study Limitations

The strengths of this study are the long follow-up periods and the large number of cases. The main limitation of our study is its retrospective nature. As a result, we could not evaluate whether the patients were using drugs that would affect hematologic parameters or if they had a disease other than malignancy affecting hematologic parameters during laboratory testing, or menopause status in women. Additionally, patients’ cytopenia status could not be evaluated by peripheral blood smear.

## Conclusion

Thrombocytopenia and lymphopenia were observed more frequently in patients older than 60 years of age and we suggest that patients of this age group who receive RAI should be surveyed particularly more carefully for these types of cytopenia. We also observed that higher doses of RAI therapy, multiple doses of RAI administration, and higher tumor burden may cause CBC abnormalities and cytopenia. The most important risk factor for lower platelet counts after RAI therapy was male gender. Clinically, the most important predictor for cytopenia is advanced disease stage, which is related to the combined effects of applied high-dose activity, multiple dose applications, and high tumor burden.

## Figures and Tables

**Table 1:**
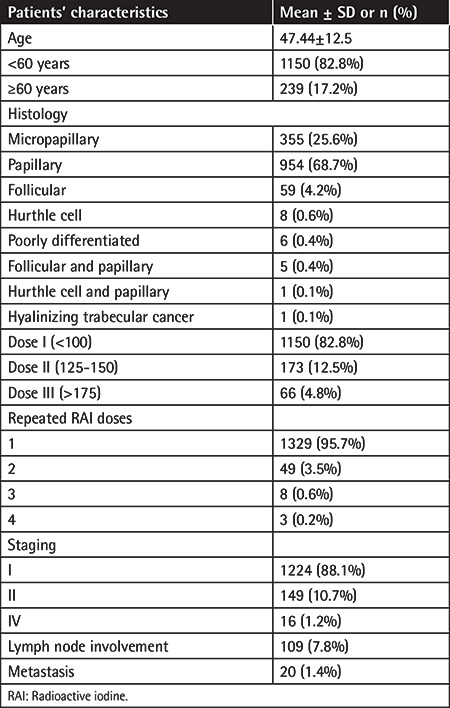
Baseline characteristics of patients.

**Table 2:**
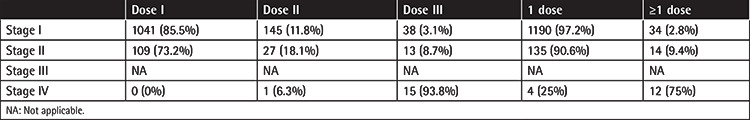
Applied doses according to stage.

**Table 3:**
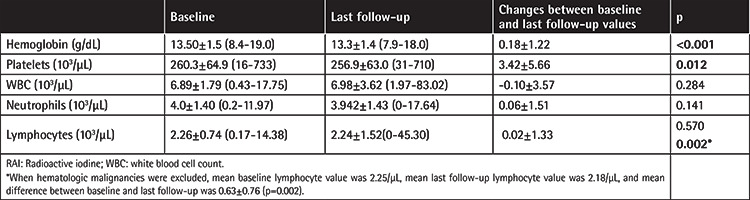
Changes in hematologic parameters with RAI therapy.

**Table 4:**
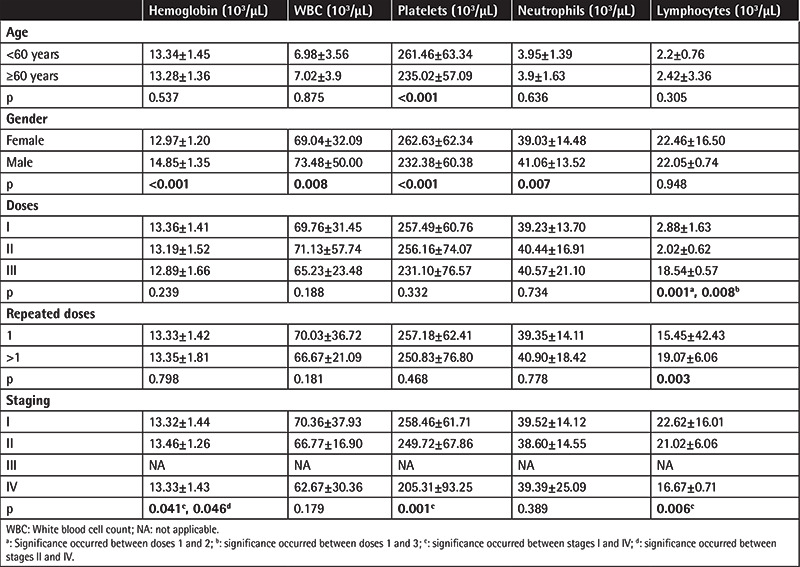
Analysis of hematologic parameters at last follow-up.

**Table 5:**
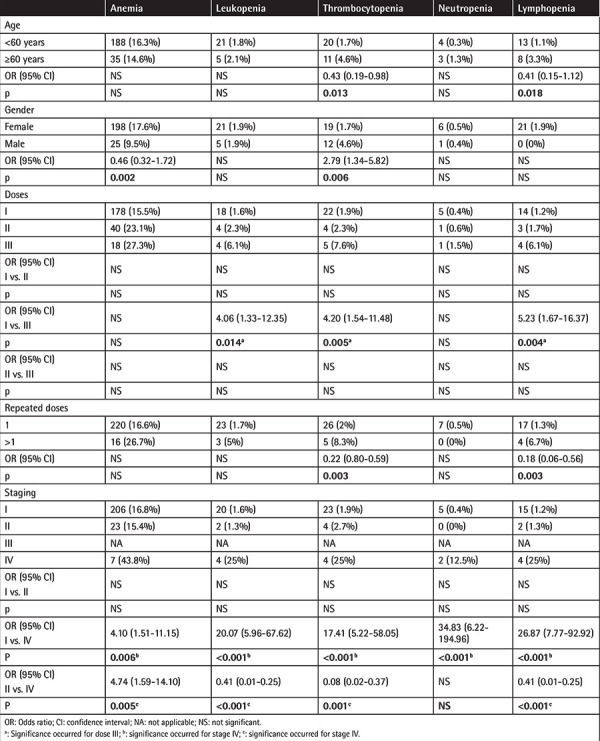
Analysis of cytopenia.

**Table 6:**
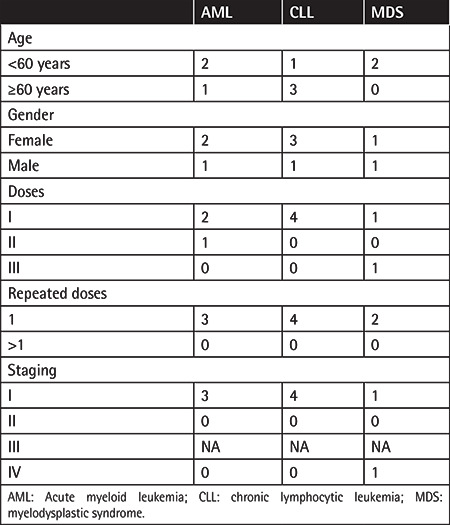
Distribution of hematologic malignancies.
